# Anti-*Helicobacter pylori* and Anti-Inflammatory Sesquiterpenoids from the Rhizoma of *Atractylodes macrocephala*

**DOI:** 10.3390/molecules30153142

**Published:** 2025-07-26

**Authors:** So Yeong Jeong, Dong-Min Kang, Hyun-Jun Kim, Sang Won Yeon, Hak Hyun Lee, Min Hee Kim, Bang Yeon Hwang, Mi-Jeong Ahn, Mi Kyeong Lee

**Affiliations:** 1 College of Pharmacy, Chungbuk National University, Cheongju 28160, Republic of Korea; wjdthdud2912@chungbuk.ac.kr (S.Y.J.); sangwon1352@chungbuk.ac.kr (S.W.Y.); leehakhyun1997@chungbuk.ac.kr (H.H.L.); smflaqh7412@chungbuk.ac.kr (M.H.K.); byhwang@chungbuk.ac.kr (B.Y.H.); 2 College of Pharmacy and Research Institute of Pharmaceutical Sciences, Gyeongsang National University, Jinju 52828, Republic of Korea; kdm7105@gnu.ac.kr; 3 Forest Medicinal Resources Research Center, National Institute of Forest Science, Yeongju 36040, Republic of Korea; mind4739@korea.kr

**Keywords:** *Atractylodes macrocephala*, anti-*Helicobacter pylori*, sesquiterpene, atractylenolide I, anti-inflammatory

## Abstract

*Helicobacter pylori*, a spiral-shaped bacterium found in the stomach, is associated with various gastrointestinal and systemic health conditions. Effective suppression of *H. pylori* is therefore critical for managing gastrointestinal diseases. In a search for natural products with anti-*H. pylori* activity, the extract of *Atractylodes macrocephala* rhizoma showed significant inhibitory effects. Chromatographic purification of *A. macrocephala* extract yielded thirteen compounds, which were identified as ten sesquiterpenes and three polyacetylenes by spectroscopic analysis. The sesquiterpene compounds belong to the eudesmane or eudesmane lactone types and exhibited structure-dependent efficacy. The major eudesmane lactone sesquiterpene, atractylenolide I (**1**), showed strong inhibitory activity comparable to metronidazole, a positive control, and atractylenolide III (**3**) also showed good efficacy. However, structural modification such as hydroxylation, methylation, or acetylation of the sesquiterpenes led to reduced activity. In contrast, polyacetylene derivatives displayed only mild inhibitory effects. Further evaluation of the active compounds against three *H. pylori* strains such as 51, 43504, and 26695 showed that atractylenolide I (**1**) had potent inhibitory effects against all three strains, with MIC_50_ values of ranging from 27.3 to 48.6 μM and MIC_90_ values from 45.4 to 87.2 μM. Atractylenolide III (**3**) exhibited selective activity against strain 51 with MIC_50_ value of 89.9 μM. Both compounds also exhibited anti-inflammatory activity with IC_90_ values of 23.3 and 31.1 μM, respectively, although they showed little effect on urease. This is the first report on the anti-*H. pylori* efficacy of various constituents of *A. macrocephala* and comparative analysis of inhibitory effects against several strains, which will provide scientific evidence supporting its potential as therapeutic agent for *H. pylori*-related infection.

## 1. Introduction

Gastric diseases, ranging from mild indigestion to stomach cancer, are prevalent worldwide and significantly impact health and quality of life. *Helicobacter pylori*, a spiral-shaped bacterium residing in the stomach, is a critical factor in the development and progression of various gastric diseases, including chronic gastritis, peptic ulcers, and stomach cancer [1,2,3]. *Helicobacter pylori* is uniquely adapted to survive in the harsh, acidic environment of the stomach. It colonizes the stomach’s mucus layer using its flagella and neutralizes gastric acid through the production of urease. These adaptations enable the bacterium to persist in the stomach, causing damage to the gastric mucosa and triggering inflammatory responses [4,5,6,7,8]. Therapies targeting *H. pylori* focus on disrupting these survival mechanisms [9,10,11]. Eradication of the bacterium remains the cornerstone of treatment, supplemented by anti-inflammatory and gastric acid-suppressive therapies. Antibiotics are the primary therapy for eradication, but antibiotic resistance poses significant challenges [12,13]. Consequently, there is an urgent need to develop new substances with anti-*H. pylori* efficacy to overcome antibiotic resistance.

Natural products, rich in diverse metabolites with unique structures, are considered valuable resources for drug discovery [14,15]. For the treatment of *H. pylori* infection, natural products hold promise for overcoming antibiotic resistance and providing synergistic effects, including anti-inflammatory benefits, through diverse mechanisms [16,17]. Efforts are also underway to enhance the effectiveness through combination of natural products with antibiotics.

*Atractylodes macrocephala*, a perennial herbaceous plant belonging to the Asteraceae family, is native to Korea, China, and Japan. It has traditionally been used as a tonic to strengthen the immune system and boost energy, as well as to treat gastrointestinal disorders such as indigestion, diarrhea, and loss of appetite [18]. A wide range of phytochemicals have been identified, including sesquiterpenoids, triterpenoids, polyacetylenes, coumarins, phenylpropanoids, flavonoids and polysaccharides [17]. Among the identified constituents, sesquiterpenes, polyacetylenes, and polysaccharides have been recognized as major components, and have been associated with a range of biological activities such as anti-inflammatory, anticancer, and antibacterial effects [19,20]. While *Atractylodes macrocephala* has been traditionally utilized for gastrointestinal ailments, its anti-*H. pylori* potential and the characterization of its active compounds have not been thoroughly investigated.

**Figure 1:**
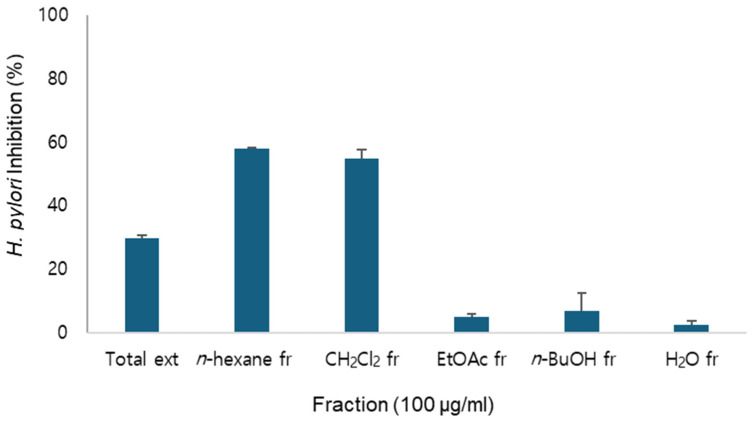
Anti-*H. pylori* activity of fractions of *A. macrocephala*.

In the screening of natural products for anti-*H. pylori* activity, the extract of *A. macrocephala* rhizoma demonstrated inhibitory activity on the growth of *H. pylori* and fractionation revealed the increased inhibitory activity on *n*-hexane and CH_2_Cl_2_ fractions (Figure 1). Therefore, this study aimed to identify the active constituents of *A. macrocephala* and evaluate their antibacterial activity against *H. pylori*. Additionally, the anti-inflammatory properties and inhibitory effects on urease were assessed to determine its potential for treating gastric disorders.

## 2. Results and Discussions

### 2.1. Anti-H.pylori Activity of A. macrocephala Extract and Fractions

As the total extract of *A. macrocephala* exhibited anti-*H. pylori* activity, identification of its active constituents was attempted. Fractionation of the total extract yielded *n*-hexane, CH_2_Cl_2_, EtOAc, *n*-BuOH and H_2_O fractions.

Among these, the *n*-hexane and CH_2_Cl_2_ fractions exhibited anti-*H. pylori* activity (Figure 1) and exhibited similar HPLC chromatograms. Therefore, they were combined for further purification.

### 2.2. Isolation of Compounds from A. macrocephala

**Figure 2:**
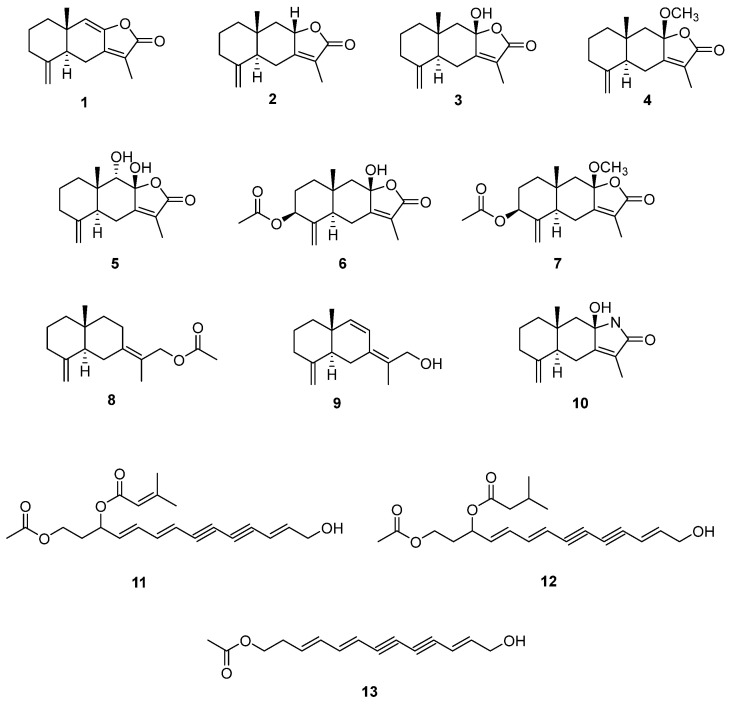
Compounds isolated from *A. macrocephala*.

Chromatographic separation led to the isolation of thirteen compounds. The structures of these isolated compounds were identified as atractylenolide I (**1**), atractylenolide II (**2**), atractylenolide III (**3**), 8β-methoxyatractylenolide (**4**), 8β,9α-dihydroxyeudesman4(15),7(11)-dien-8,12-olide (**5**), atractylenolide IV (**6**), 9α-methoxy-3,8-dimethyl-5-methylene-2-oxo-2,4,4α,5,6,7,8,8α,9,9α-decahydronaphtho [2,3-β]furan-6-yl acetate (**7**), atractylenolide VII (**8**), atractylmacrols D (**9**), taenialactam B (**10**), 14-acetoxy-12-senecioyloxytetradeca-2*E*,8*E*,10*E*-trien-4,6-diyn-1-ol (**11**), 14-acetoxy-12-methylbutyl-2*E*,8*E*,10*E*-trien-4,6-diyn-1-ol (**12**) and 4,6,12-tetradecatriene-8,10-diyn-1-ol,1-acetate (**13**) by the comparison with previously published data (Figure 2) [21,22,23,24,25,26,27]. These compounds are ten sesquiterpenes (**1**–**10**) and three polyacetylenes (**11**–**13**), which have been reported previously from *Atractylodes* species.

### 2.3. Anti-H. pylori Activity of Compounds of A. macrocephala Rhizoma

#### 2.3.1. *H. pylori* Inhibitory Activity

**Figure 3:**
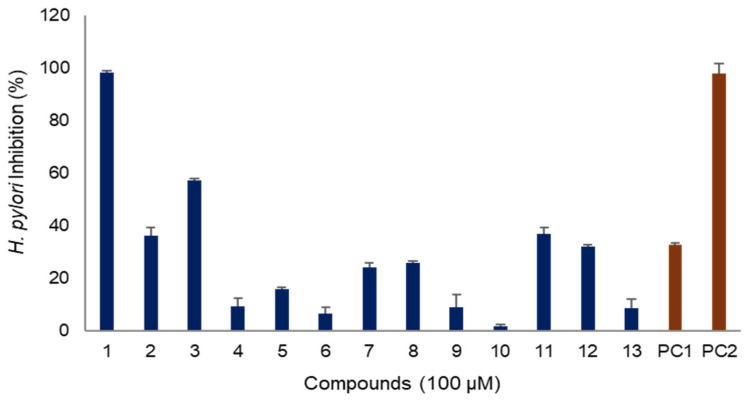
Anti-*H. pylori* activity of compounds isolated from *A. macrocephala*. PC: positive control (PC1: quercetin, PC2: metronidazole).

The anti-*H. pylori* activity of the isolated compounds was evaluated (Figure 3). Among the compounds derived from *A. macrocephala*, atractylenolide I (**1**) exhibited the best efficacy, with an inhibitory effect of 98% at a concentration of 100 μg/mL, similar to that of metronidazole, a positive control used as an antibiotic in clinical field. Atractylenolide III (**3**) also demonstrated an inhibitory efficacy of 57% at the same concentration. However, other sesquiterpenes exhibited weaker efficacy, with inhibition rates of less than 30%. Additionally, the polyacetylenes, 14-acetoxy-12-senecioyloxytetradeca-2*E*,8*E*,10*E*-trien-4,6-diyn-1-ol (**11**) and 14-acetoxy-12-methylbutyl-2*E*,8*E*,10*E*-trien-4,6-diyn-1-ol (**12**), showed anti-*H. pylori* activities of 37% and 32%, respectively, which were comparable to the activity of quercetin, a natural compound used as a positive control (Figure 3).

#### 2.3.2. Structure Activity Relationship for *H. pylori* Inhibitory Activity

The compounds isolated from *A. macrocephala* in this study can be categorized into sesquiterpenes (**1**–**10**) and polyacetylenes (**11**–**13**). The anti-*H. pylori* activity exhibited by these compounds was highly dependent on their structural features. All the sesquiterpenes isolated in this study share eudesmane skeleton, which can be further divided into eudesmanes (**8**–**10**) and eudesmane lactones (**1**–**7**) based on the presence of lactone moiety in the structure. Each sesquiterpene differs in the substituents, such as double bonds, hydroxyl (OH), methoxyl (OCH_3_), and acetyl (CH_3_CO) groups, as well as their positions, which significantly affected their efficacy. Compounds **1**–**7** belong to eudesmane lactone category. Among them, atractylenolide I (**1**), which possesses an additional double bond, showed the best efficacy, whereas atractylenolide II (**2**), with the same structure as atractylenolide I (**1**) but lacking a double bond, exhibited relatively mild efficacy. Atractylenolide III (**3**), which showed excellent efficacy, has hydroxyl moiety at C-8. The efficacy decreased when there was no hydroxyl moiety, as in atractylenolide II (**2**), or when the hydroxyl moiety was substituted with methoxyl, as in 8β-methoxyatractylenolide (**4**). The addition of hydroxyl or acetyl moieties, as seen in 8β,9α-dihydroxyeudesman4(15),7(11)-dien-8,12-olide (**5**) and atractylenolide IV (**6**), reduced efficacy. Moreover, replacement of oxygen of lactone with nitrogen also reduced efficacy. Additionally, eudesmane lactone sesquiterpenes were more effective than eudesmane without a lactone moiety. Based on our structure-activity relationship, it was confirmed that eudesmane lactone sesquiterpenes hold the potential for anti-*H. pylori* efficacy, though there are considerable differences depending on their structures. In the case of polyacetylenes, compounds with acetyl and prenyl moieties exhibited mild efficacy. Therefore, it is presumed that these compounds may contribute synergistically to the overall anti-*H. pylori* activity of *A. macrocephala*.

#### 2.3.3. Minimum Inhibitory Concentrations (MIC) Against Different *H. pylori* Strains

The MICs of atractylenolide I (**1**) and atractylenolide III (**3**), which exhibited excellent efficacy, were determined for several *H. pylori* strains 51, 26695, and 43504, as shown in Table 1. Atractylenolide I (**1**) showed excellent inhibitory efficacy against all strains. In particular, atractylenolide I (**1**) showed the most potent anti-*H. pylori* activity against strain 51 with MIC_50_, MIC_90_ and MBC values of 27.3, 45.8 and 54.9 μg/mL, respectively. These values were comparable to the antibiotic metronidazole, an antibiotic in clinical field, which showed MIC_50_, MIC_90_ and MBC values of 14.9, 49.8, and 59.7 μM, respectively. Atractylenolide I (**1**) also showed potent anti-*H. pylori* activity against strains 26695 and 43504, with MIC_50_ values of 43.3 and 48.6 μg/mL, MIC_90_ values of 79.1 and 97.4 μg/mL and MBC values of 91.9 and 94.3 μg/mL, respectively. The anti-*H. pylori* activity of atractylenolide III (**3**) was weaker compared to atractylenolide I (**1**) but still showed lower MIC values than quercetin, a positive control derived from natural product.

#### 2.3.4. Urease Inhibitory Activity

The virulence factors of *H. pylori* include motility, exotoxin, mucinase, adhesion, and urease activity [28]. The acid tolerance of *H. pylori* is primarily attributed to its urease activity, an enzyme found in the cytoplasm. Urease produced by *H. pylori* initiates hydrolysis of urea, resulting in ammonia production, which creates an alkaline environment in the stomach. This alkaline condition supports the survival of *H. pylori* and further triggers inflammation and various diseases. Urease inhibitors are therefore used in clinical therapy [29]. The inhibitory effects of atractylenolide I (**1**) and atractylenolide III (**3**) on urease activity were evaluated, but no inhibition was observed in our assay system (Table 2).

### 2.4. Anti-Inflammatory Activity

Inflammation is a key pathological characteristic of *H. pylori* infection. Persistent infection with *H. pylori* leads to inflammation through the activation of macrophages, resulting in damage to the gastric mucosa and various gastric diseases. Therefore, anti-inflammatory therapy can help alleviate the damages caused by *H. pylori* infection. Therefore, anti-inflammatory activity of atractylenolide I (**1**) and atractylenolide III (**3**) were evaluated. Both compounds showed anti-inflammatory activity in LPS-stimulated RAW 264.7 macrophage cells (Table 3). The IC_50_ values of atractylenolide I (**1**) and atractylenolide III (**3**) were 23.3 and 31.2 μM, respectively, showing comparable activity to the positive control, aminoguanidine, which had an IC_50_ of 24.6 μM. 

### 2.5. Potential as Treatment for H. pylori Infection

*Atractylodes* species are well-recognized for their immunomodulatory effects in traditional medicine [30,31]. Notably, their essential oil components have been traditionally utilized for the improvement and treatment of gastrointestinal disorders. These oils have demonstrated anti-inflammatory and antioxidant properties, contributing to the prevention and treatment of gastric ulcers and gastric cancer [19]. Additionally, essential oils from *Atractylodes* spp. exhibit antibacterial and antiviral activities, including reported efficacy against methicillin-resistant *Staphylococcus aureus* (MRSA) and suppression of SARS-CoV-2 replication [32,33]. However, their inhibitory activity against *H. pylori* has not yet been reported.

In this study, we evaluated the anti-*H. pylori* activity of *A. macerophala* extract and investigated the activity of their major bioactive components. Thirteen compounds, including ten sesquiterpenes and three polyacetylenes, were isolated. Although the compounds were previous reported, the anti-*H. pylori* activity of these compounds have not been previously reported. Therefore, their anti-*H. pylori* activity were comparatively analyzed and structure-activity relationship was suggested. The major compound of *A. macerophala* exhibited strong *H. pylori* inhibitory activity and our study also demonstrated anti-H. pylori activity against multiple *H. pylori* strains which support its broad spectrum efficacy for its potential therapeutic application. To better understand the mechanism of action, urease inhibition was examined. Interestingly, while the major constituents exhibited strong anti-*H. pylori* effects, they did not show urease inhibition activity, implying a non-urease-related mechanism of action such as the inhibition of *H. pylori* virulence factors including CagA and VacA. They also exhibited strong anti-inflammatory effects, which suggested the potential of *A. macerophala* as an effective treatment for *H. pylori* infection, potentially offering a synergistic effect due to its combined anti-*H. pylori* and anti-inflammatory properties. Taken together our present study provides the scientific supports for therapeutic effects of *A. macerophala* for gastrointestinal diseases through mechanisms targeting *H. pylori*.

## 3. Materials and Methods

### 3.1. Plant Material

The rhizoma of *A. macrocephala* was sourced from the National Institute of Forest Science (Yeongju, Korea). After identification by the Herbarium of the College of Pharmacy at Chungbuk National University, voucher specimens (CBNU2023-AM) were deposited in the herbarium’s specimen repository.

### 3.2. General Experimental Procedure

The NMR signals were analyzed using a Bruker DRX 400 MHz spectrometer (Bruker-Biospin, Karlsruhe, Germany), with methanol-d4 serving as the solvent. The UV and IR spectra were acquired using the Jasco UV-550 (JASCO, Tokyo, Japan) and Perkin–Elmer model LE599 (Perkin–Elmer, Waltham, MA, USA) spectrometers. The semi-preparative high-performance liquid chromatography (HPLC) was conducted using a Waters 515 HPLC pump (Waters Corp., Milford, MA, USA) equipped with a 996 photodiode array detector and controlled by Waters Empower software (Version 3.8.0). The chromatographic separation was achieved using a Gemini-NX ODS column with dimensions of 150 × 10.0 mm and 150 × 21.2 mm. The experiment included using aluminum plates that were pre-coated with Kieselgel 60 F254 (0.25 mm, Merck, Darmstadt, Germany) to conduct thin-layer chromatography (TLC).

### 3.3. Extraction and Fractionation

For the preparation of extract, the dried powder of *A. macrocephala* rhizoma (2.2 kg) was extracted with 80% MeOH (20 L × 2) at room temperature. The 80% MeOH extract (560.0 g) was suspended in water and partitioned sequentially with *n*-hexane, CH_2_Cl_2_, EtOAc, and *n*-BuOH (Duksan, Seoul, Korea). These extract and fractions were used for evaluation of activity.

### 3.4. Isolation of Compounds

As the *n*-hexane and CH_2_Cl_2_ fractions showed similar HPLC chromatograms, they were combined for further purification. The combined fraction (AMC, 46.3 g) was chromatographed on silica gel eluted with a mixture of CH_2_Cl_2_-MeOH (100:0 to 0:100, gradient), yielding five subfractions (AMC1–5). Subfraction AMC1 was further separated by MPLC on silica gel with a mixture of *n*-hexane-CH_2_Cl_2_ (100:0 to 0:100, gradient), producing six subfractions (AMC1A–F). Subfraction AMC1D was subjected to RP silica MPLC using MeCN-H_2_O (20:80), yielding nine subfractions (AMC1D1–9). Compounds **1** and **5** were purified from AMC1D5 by semi-preparative HPLC (MeCN-H_2_O, 53:47), while compounds **8** and **9** were obtained from AMC1D7 and AMC1D8, respectively, using MeCN-H_2_O (30:70). Subfraction AMC1E was purified with RP silica MPLC using MeCN-H_2_O (50:50) to yield eight subfractions (AMC1E1–8). Compounds **11**, **12**, and **13** were purified from AMC1E7 by semi-preparative HPLC (MeCN-H_2_O, 65:35). Subfraction AMC2 was chromatographed on silica gel with a mixture of CH_2_Cl_2_-MeOH (100:0 to 0:100, gradient), yielding five subfractions (AMC2A–E). Subfraction AMC2B was further processed with MPLC on silica gel using a mixture of *n*-hexane-EtOAc (100:0 to 0:100, gradient), producing seven subfractions (AMC2B1–7). Subfraction AMC2B3 was subjected to RP silica MPLC with MeCN-H_2_O (50:50), yielding nine subfractions (AMC2B3A–I). Compounds **2** and **10** were purified from AMC2B3D and AMC2B3H, respectively, by semi-preparative HPLC (MeCN-H_2_O, 60:40). Subfraction AMC3, separated on silica gel with a CH_2_Cl_2_-MeOH gradient (100:0 to 0:100), yielded five subfractions (AMC3A–E). Subfraction AMC3C was further separated with RP silica MPLC using MeCN-H_2_O (50:50) to obtain five subfractions (AMC3C1–5). Compound 6 was purified from AMC3C2 by semi-preparative HPLC (MeCN-H_2_O, 30:70), while compounds **3**, **4**, and **7** were isolated from AMC3C4 using MeCN-H_2_O (35:65).

### 3.5. Identification of Structures

The structures of the isolated compounds were elucidated based on spectroscopic analyses, including NMR and MS, and confirmed by comparison with published data. The purity of the compounds was assessed using HPLC and NMR. The NMR spectra of compounds **1**–**13** are provided in the Supplementary Information.

### 3.6. Helicobacter pylori Culture

The *H. pylori* strains 51, 26695, and 43504 were obtained from the Korean Type Culture Collection, Korea. Strain 51 was originally isolated from the stomach of a Korean patient in 1987, while strain 43504 was isolated from the stomach of an American patient in 2008. The strain 26695, also known as KE26695 was identical with the strain isolated in the United Kingdom from the stomach of a patient with gastritis. All strains were cultured and maintained on Brucella agar medium (BD Co., Sparks, MD, USA) supplemented with 10% horse serum (Gibco, New York, NY, USA). The cultures were incubated at 37 °C under conditions of 100% humidity and 10% CO_2_. Subculturing was performed every 2–3 days to maintain viable cultures.

### 3.7. MICs and MBC Determination

Minimal inhibitory concentrations (MICs) were determined with the broth dilution method, as shown in our previous report [34]. After incubation for 24 h, growth was evaluated by reading optical density at 600 nm. MIC_50_ and MIC_90_ were defined as the lowest concentration of inhibiting growth by 50 and 90%, respectively. DMSO was used as the negative control, while quercetin and metronidazole were used as positive controls. Minimum bactericidal concentration (MBC) was determined by re-culturing broth dilution which inhibited the growth of *H. pylori* on the agar plate according to our previous report [35]. The broth dilution was streaked onto Brucella agar plate and incubated for 48 h. The MBC value was defined as the lowest concentration at which no bacterial colonies appeared on agar plates.

### 3.8. Urease Inhibitory Activity

Urease inhibitory activity was assessed using phenol red reagent, as described in a previously reported study [36]. The activity of the uninhibited enzyme was used as a control, with an enzyme activity value set at 100%. The inhibitory activity of compounds against *H. pylori* urease was calculated as [(absorbance of control—absorbance of solution with samples)/absorbance of control] × 100.

### 3.9. Measurement of LPS-Induced NO Production

RAW 264.7 macrophages cells were obtained from the American Type Culture Collection (Manassas, VA, USA), and cultured in Dulbecco’s modified Eagle’s medium (DMEM, Gibco-BRL., St. Louis, MO, USA) containing 10% heat-inactivated fetal bovine serum and penicillin/streptomycin (100 U/mL). The inhibitory effect of compounds on nitric oxide (NO) production was evaluated using RAW 264.7 cells induced by lipopolysaccharide (LPS) [37]. RAW 264.7 cells were treated with 1 μg/mL LPS in the presence or absence of compounds. After 24 h incubation, the cell medium was mixed with Griess reagent and the amount of NO produced was determined by measuring the absorbance at 550 nm using an ELISA reader (Molecular Device, San Jose, CA, USA). Cell viability was assessed by MTT assay.

### 3.10. Statistical Analysis

Data were presented as means ± standard deviations of two or triple independent experiments. Statistical significance (*p* < 0.05) was assessed by one-way analysis of variance (ANOVA) using coupled with Dunnett’s *t*-tests SPSS Statistics 24.0 software (IBM, Armonk, NY, USA). GraphPad Prism Version 5.01 (GraphPad Software, Inc., San Diego, CA, USA) was used to calculate the MIC_50_ and MIC_90_ values. The values were obtained from triplicate determinations and two independent experiments.

## 4. Conclusions

*A. macerophala* extract demonstrated the efficacy of anti-*H. pylori*; therefore, its bioactive compounds were isolated through chromatographic techniques and identified by spectroscopic analysis. Thirteen compounds were isolated, with two major sesquiterpenes, atractylenolide I (**1**) and atractylenolide III (**3**), exhibiting excellent anti-*H. pylori* efficacy, while polyacetylene showed mild activity. Atractylenolide I (**1**) and atractylenolide III (**3**) did not inhibit urease, but exhibited strong anti-inflammatory effects. Therefore, *A. macerophala* holds potential as an effective treatment for *H. pylori* infection, potentially offering a synergistic effect due to its combined anti-*H. pylori* and anti-inflammatory properties.

## Figures and Tables

**Table 1:** Anti-*H. pylori* activity of atractylenolide I (**1**) and atractylenolide III (**3**) of *A. macrocephala*. PC: positive control (PC1: metronidazole, PC2: quercetin).

Strains	MIC (μg/mL)	Atractylenolide I (1)	Atractylenolide III (3)	PC1	PC2
51	MIC	3.13	12.5	3.13	50
	MIC_50_	27.3	89.9	14.9	>100
	MIC_90_	45.8	>100	49.8	>100
	MBC	54.9	>100	59.7	>100
26695	MIC	6.25	25	3.13	50
	MIC_50_	43.3	>100	20.9	>100
	MIC_90_	79.1	>100	42.1	>100
	MBC	91.9	>100	53.1	>100
43504	MIC	12.5	100	3.13	50
	MIC_50_	48.6	>100	28.3	>100
	MIC_90_	87.2	>100	62.1	>100
	MBC	94.3	>100	81.5	>100

**Table 2:** Urease inhibitory activity of atractylenolide I (**1**) and atractylenolide III (**3**).

	Urease Inhibition (%)		
	250 μM	500 μM	1000 μM
atractylenolide I (**1**)	0.1 ± 4.4	5.8 ± 5.3	5.7 ± 3.2
atractylenolide III (**3**)	1.9 ± 5.0	2.2 ± 3.4	3.2 ± 5.1

**Table 3:** Anti-inflammatory activity of atractylenolide I (**1**) and atractylenolide III (**3**) in LPS-induced RAW264.7 cells.

	Inhibition on NO Production (%)			IC_50_ (μM)
	10 μM	25 μM	50 μM	
atractylenolide I (**1**)	29.6 ± 1.66	54.0 ± 3.00	89.9 ±0.99	23.3
atractylenolide III (**3**)	23.0 ± 3.30	43.6 ± 4.11	71.7 ± 4.33	31.1

## Data Availability

The data will be made available on request.

## Supplementary Materials

The following supporting information can be downloaded at https://www.mdpi.com/article/10.3390/molecules30153142/s1, Figures S1–S26: NMR spectrum of compounds **1**–**13**.
