# Supplementary material for: Anti-Helicobacter pylori and Anti-Inflammatory Sesquiterpenoids from the Rhizoma of Atractylodes macrocephala
Source: Molecules. 2025 Jul 26;30(15):3142. doi: 10.3390/molecules30153142 (PMC12348959; doi:10.3390/molecules30153142)
Supplement: Supplementary file 1 [file molecules-30-03142-s001.zip › molecules-3745127-supplementary.pdf]

Supplementary Information

Anti-*Helicobacter pylori* and Anti-Inflammatory Sesquiterpenoids from the Rhizoma of *Atractylodes macrocephala*

So Yeong Jeong [1,†], Dong-Min Kang [2,†], Hyun-Jun Kim [3], Sang Won Yeon [1], Hak Hyun Lee [1], Min Hee Kim [1], Bang Yeon Hwang [1], Mi-Jeong Ahn [2,*] and Mi Kyeong Lee [1,*]

[1] College of Pharmacy, Chungbuk National University, Cheongju 28160, Republic of Korea; wjdthdud2912@chungbuk.ac.kr (S.Y.J.); sangwon1352@chungbuk.ac.kr (S.W.Y.); leehakhyun1997@chungbuk.ac.kr (H.H.L.); smflaqh7412@chungbuk.ac.kr (M.H.K.); byhwang@chungbuk.ac.kr (B.Y.H.)

[2] College of Pharmacy and Research Institute of Pharmaceutical Sciences, Gyeongsang National University, Jinju 52828, Republic of Korea; kdm7105@gnu.ac.kr

[3] Forest Medicinal Resources Research Center, National Institute of Forest Science, Yeongju 36040, Republic of Korea; mind4739@korea.kr

* Correspondence: amj5812@gnu.ac.kr (M.-J.A.); mklee@chungbuk.sv.kr (M.K.L.)

† These authors contributed equally to this work.

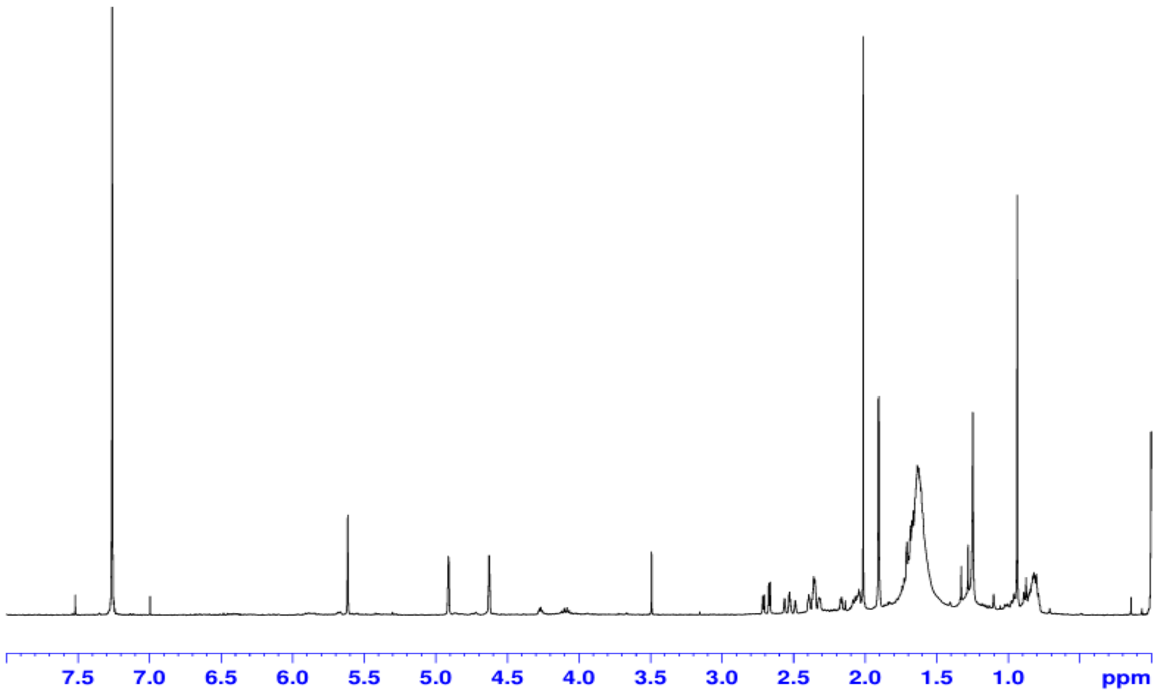

Figure S1. ^{1}H NMR spectrum of compound **1**

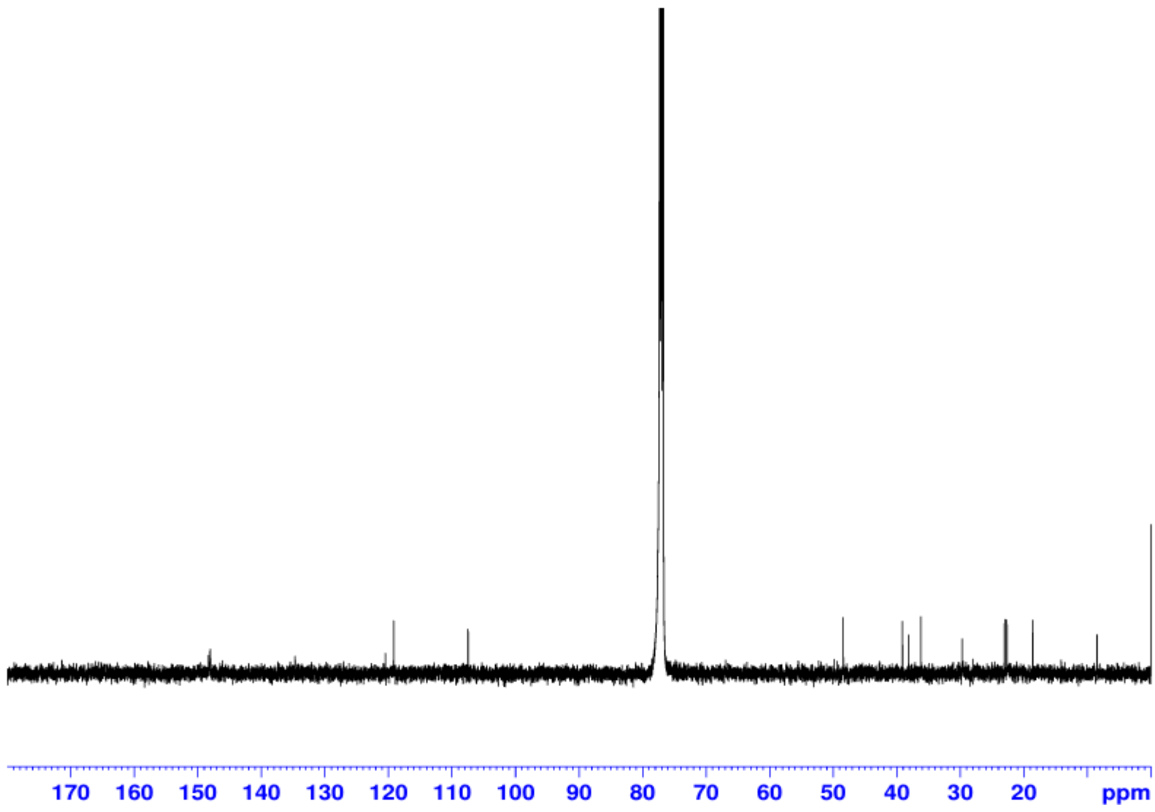

Figure S2. ^{13}C NMR spectrum of compound **1**

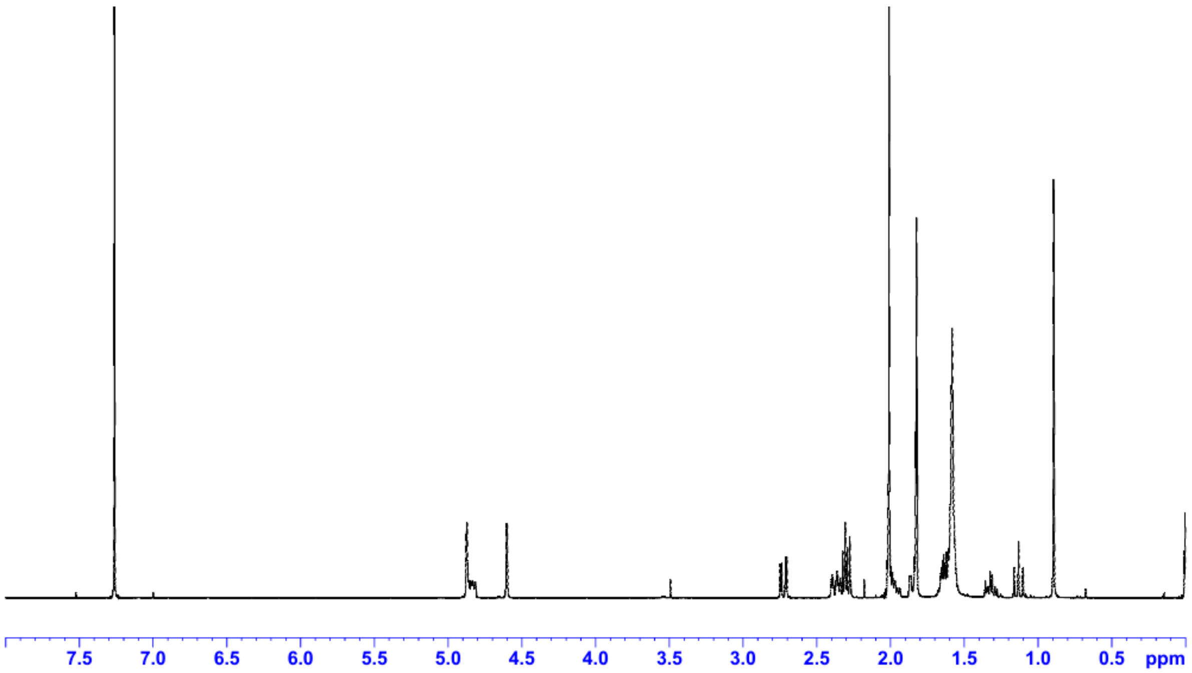

Figure S3. ^{1}H NMR spectrum of compound **2**

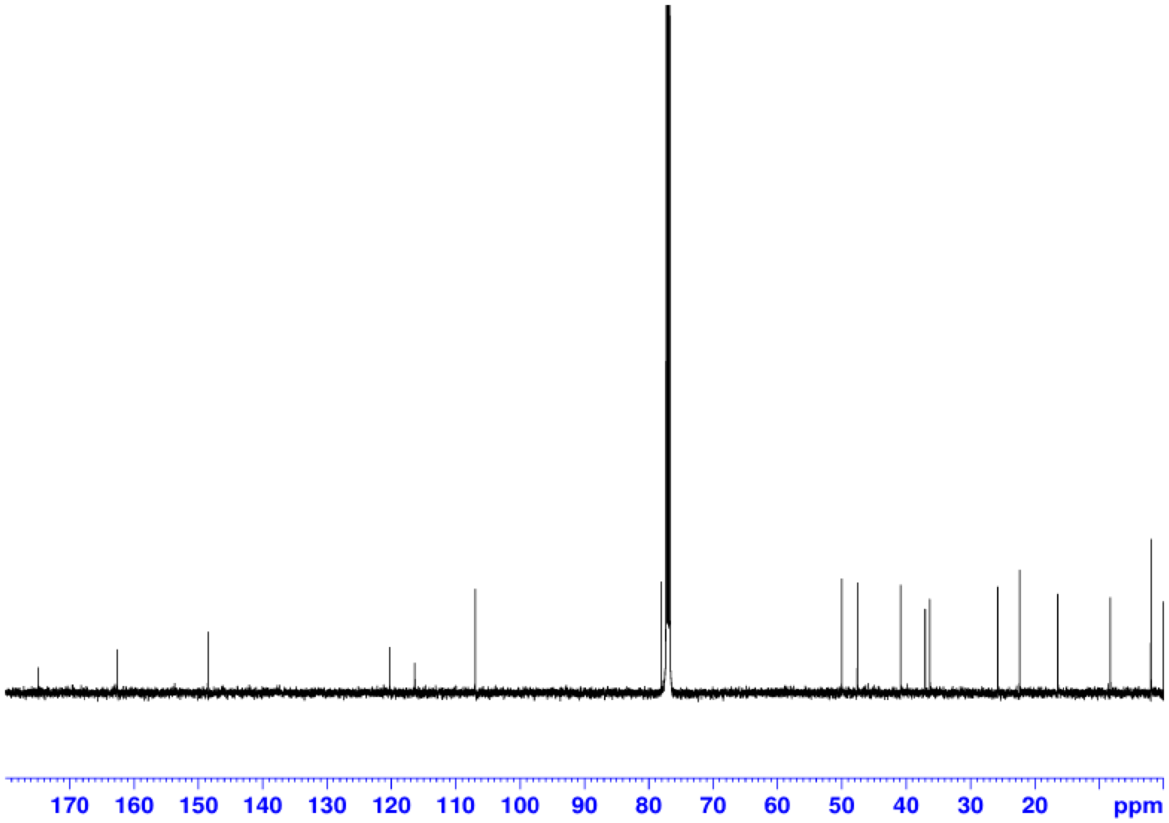

Figure S4. ^{13}C NMR spectrum of compound **2**

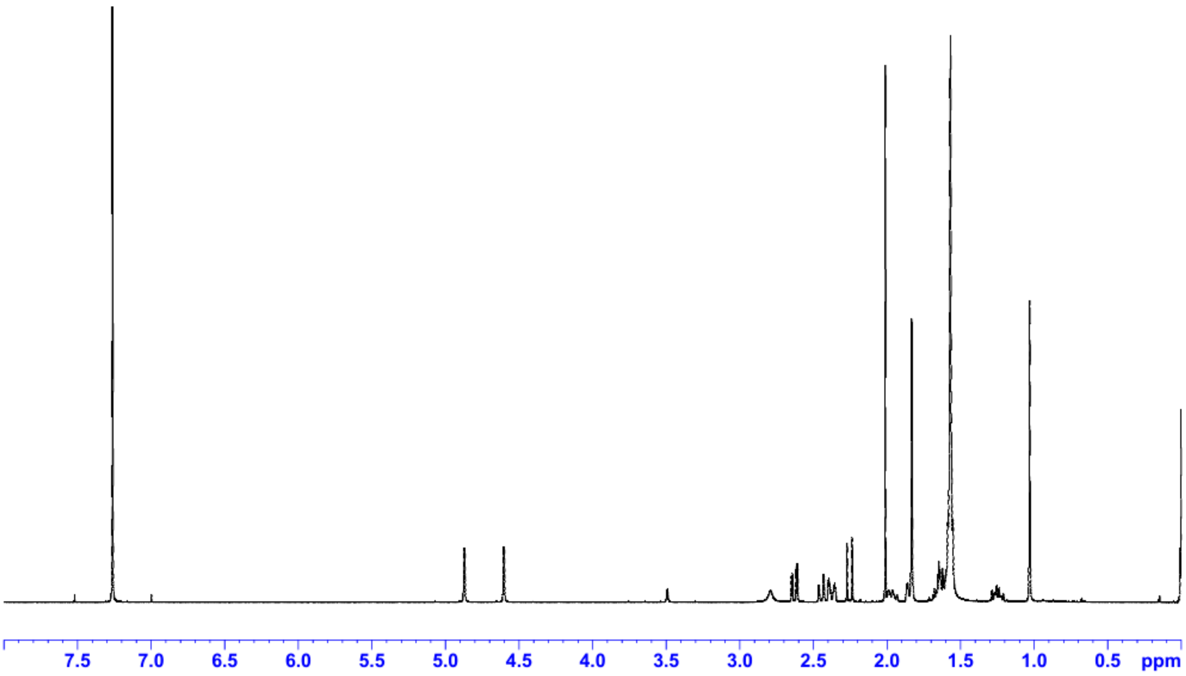

Figure S5. ^{1}H NMR spectrum of compound **3**

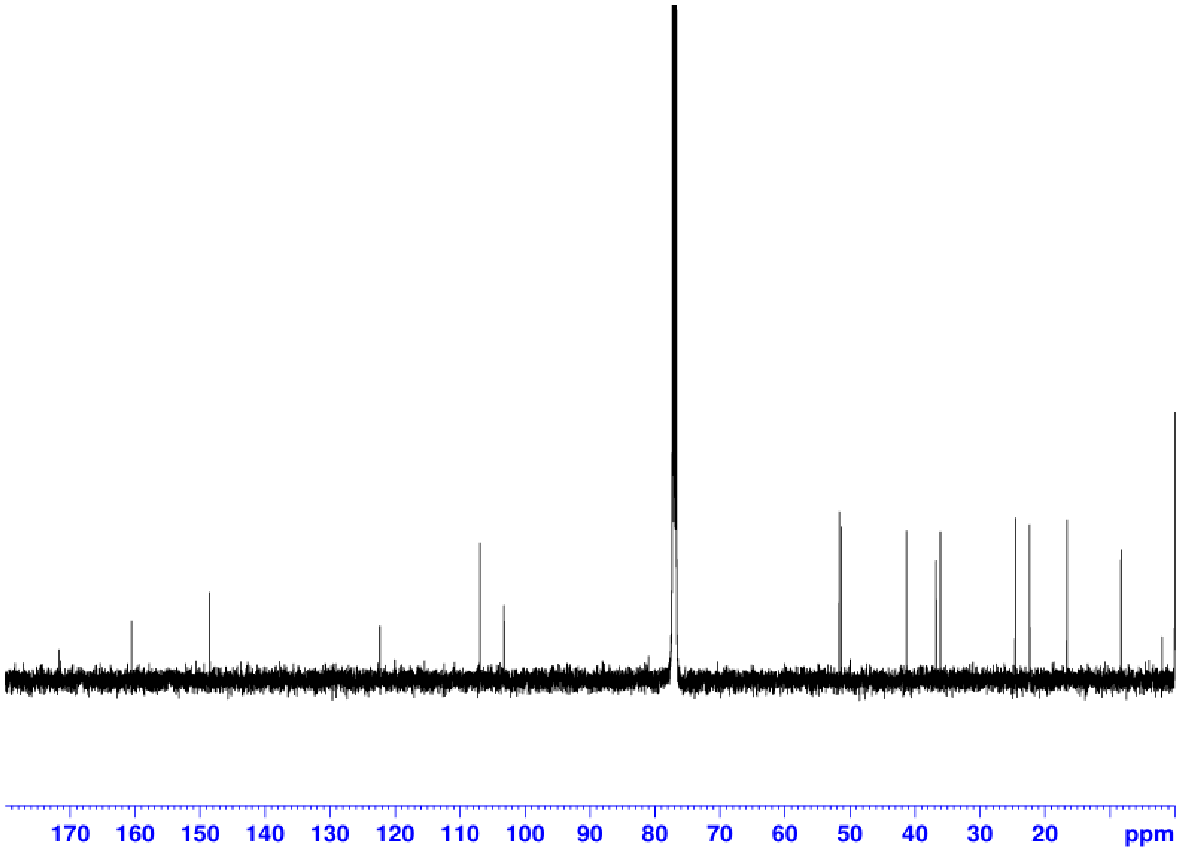

Figure S6. ^{13}C NMR spectrum of compound **3**

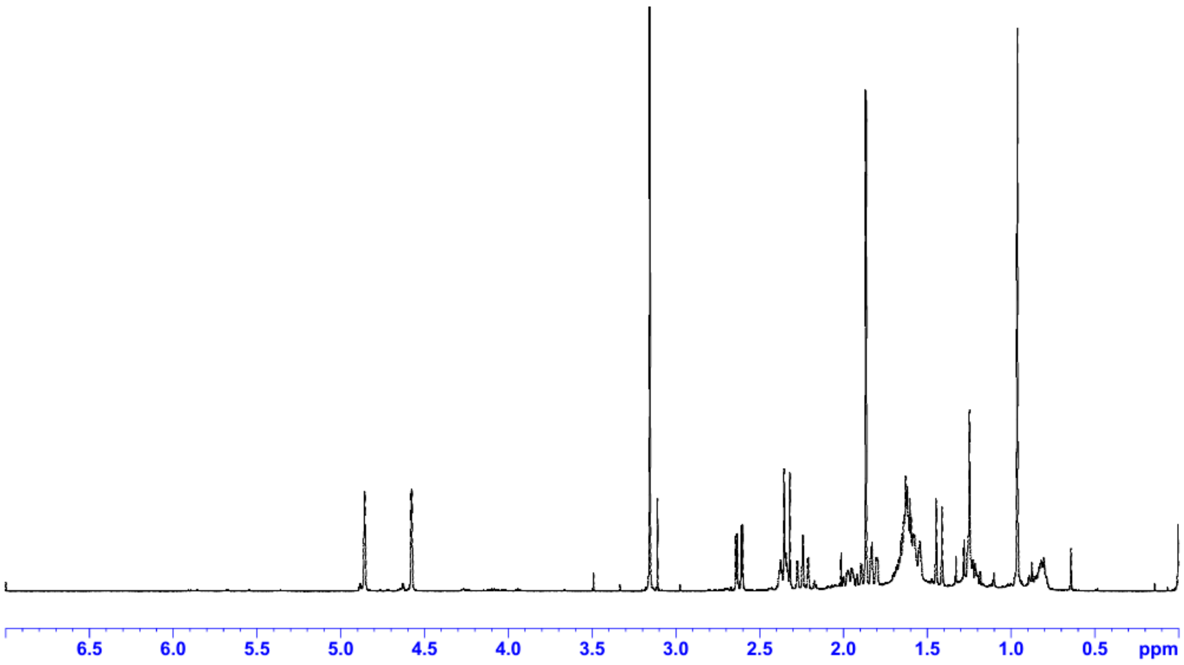

Figure S7. ^{1}H NMR spectrum of compound **4**

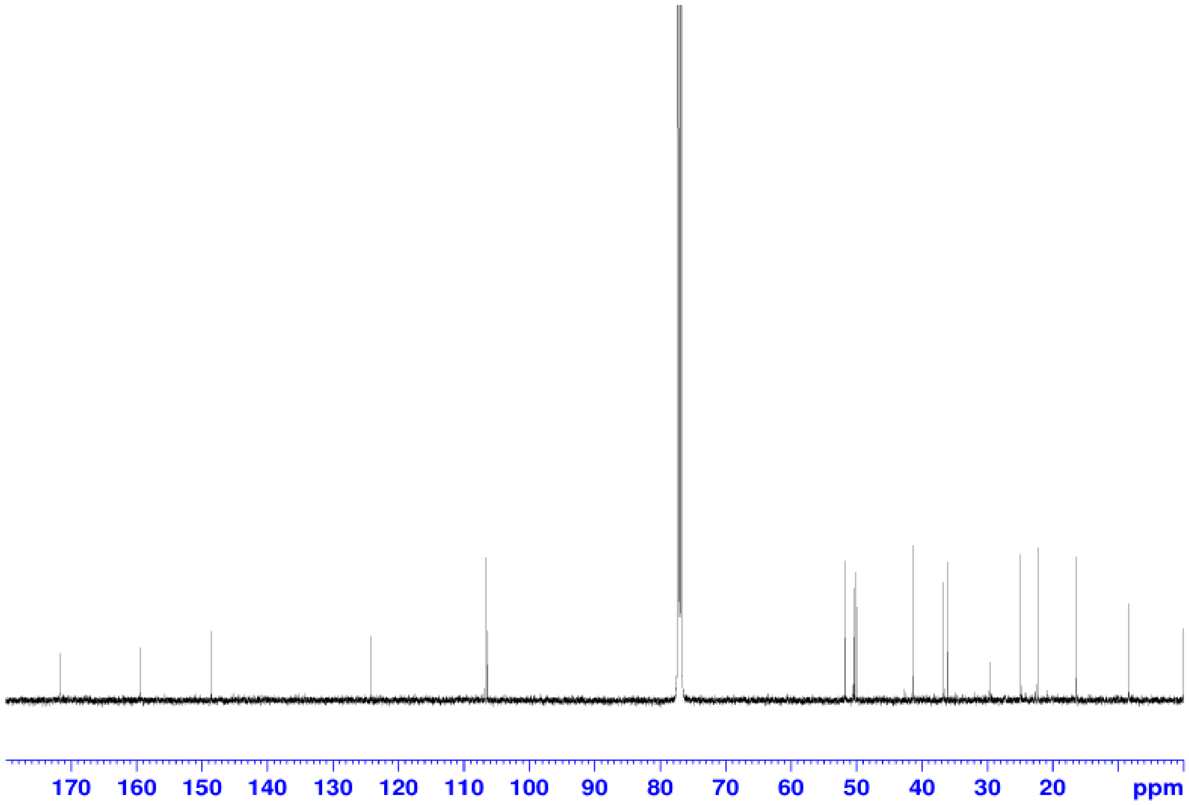

Figure S8. ^{13}C NMR spectrum of compound **4**

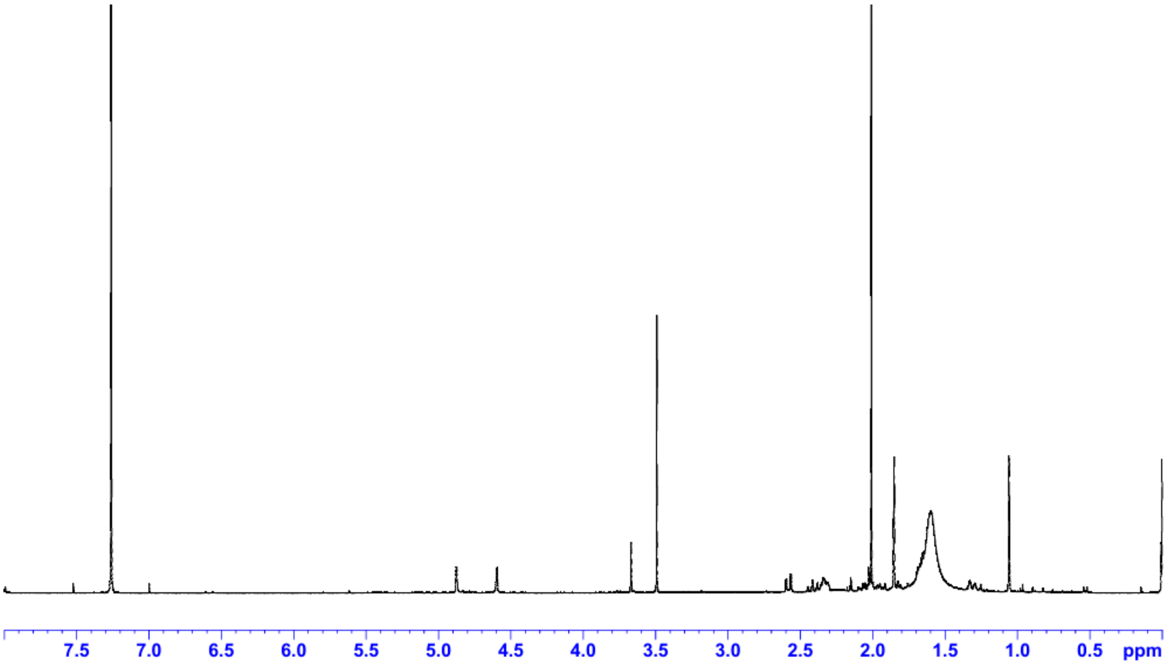

Figure S9. ^{1}H NMR spectrum of compound **5**

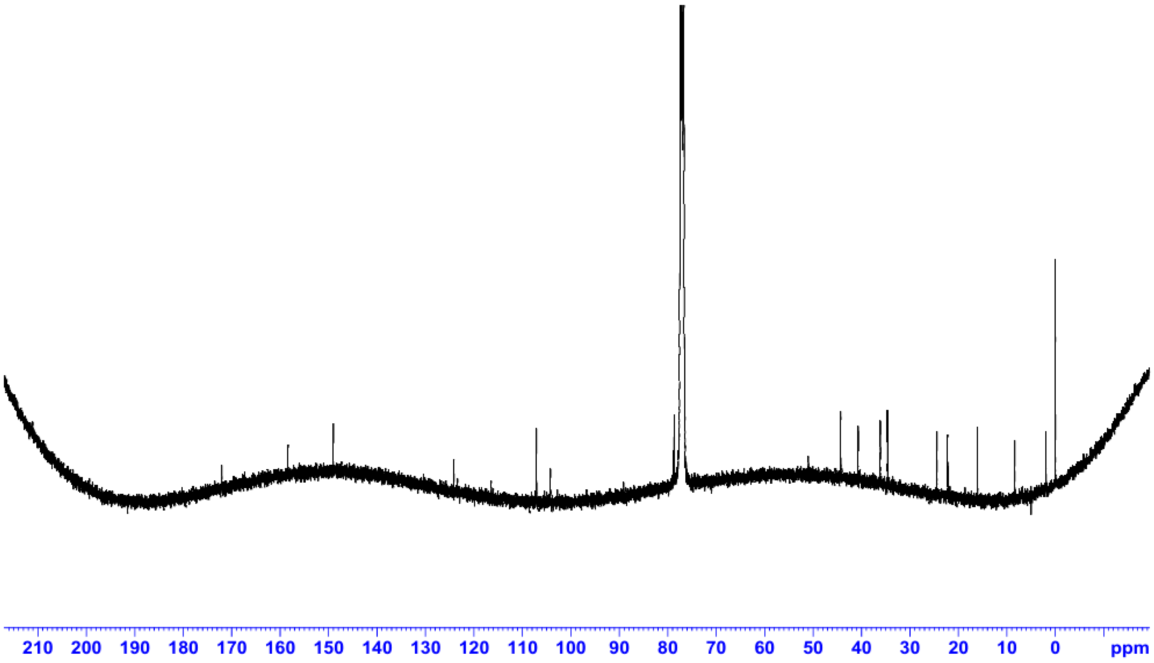

Figure S10. ^{13}C NMR spectrum of compound **5**

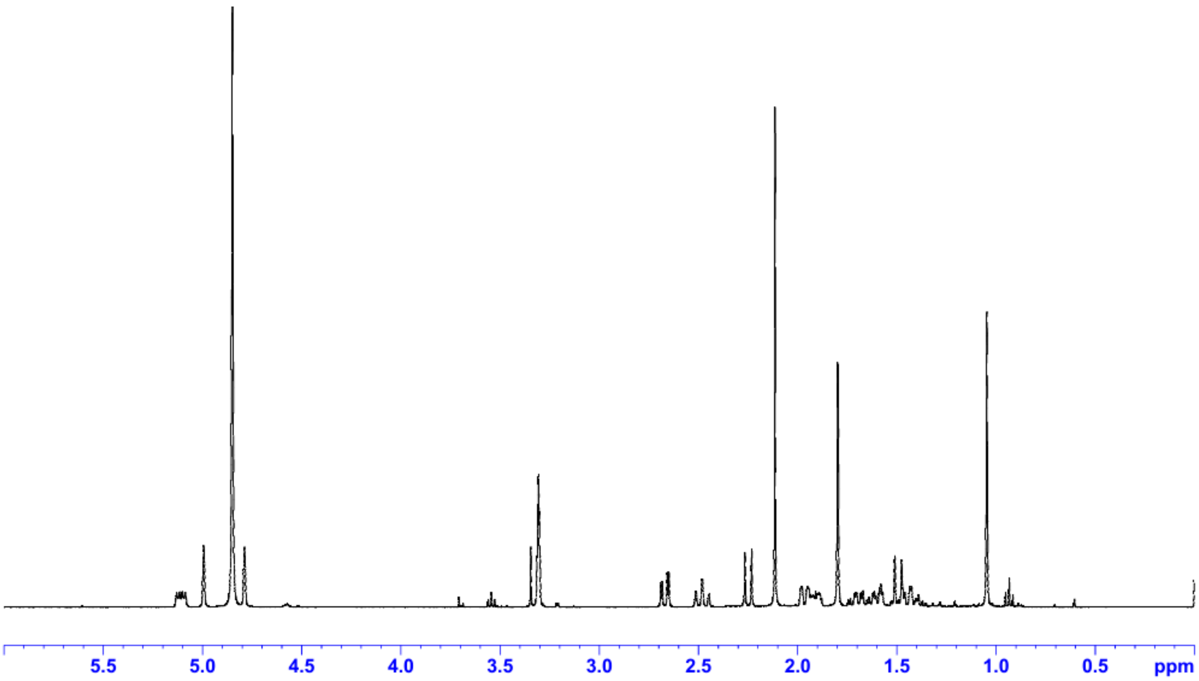

Figure S11. ¹H NMR spectrum of compound **6**

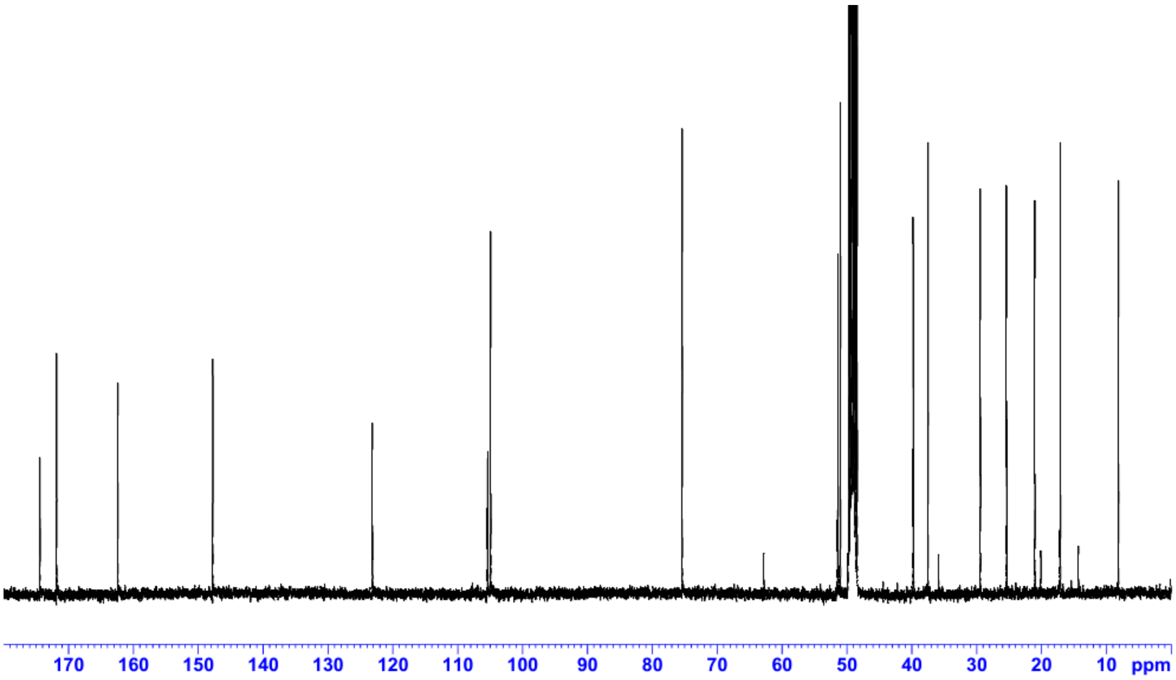

Figure S12. ¹³C NMR spectrum of compound **6**

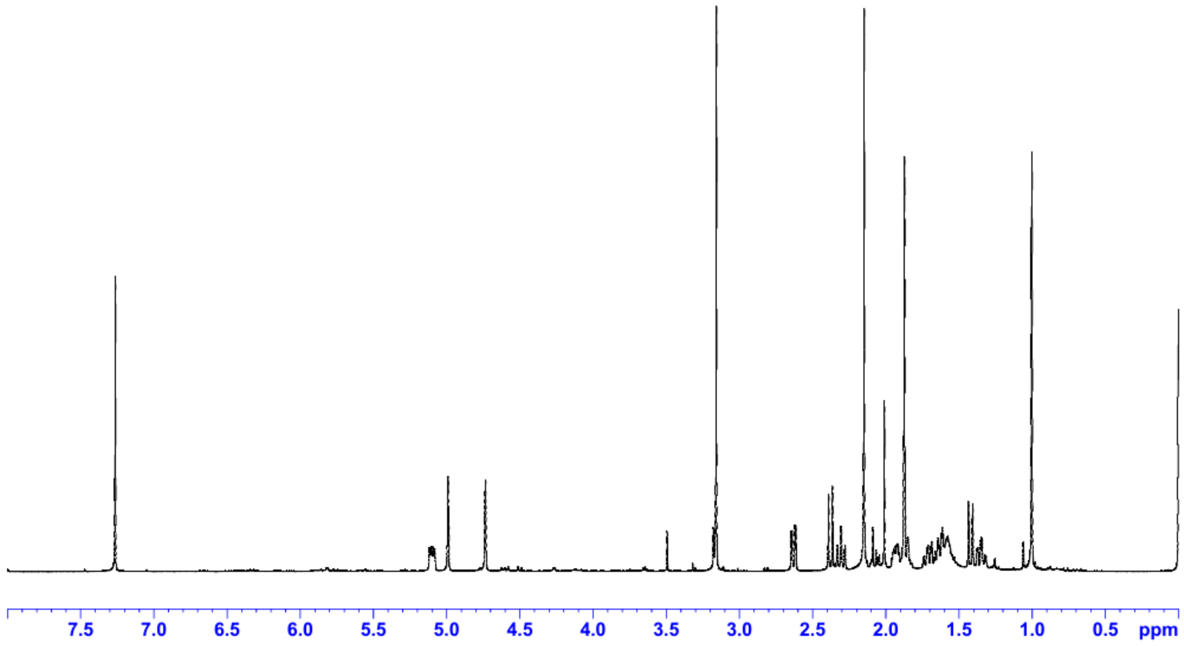

Figure S13. ^{1}H NMR spectrum of compound **7**

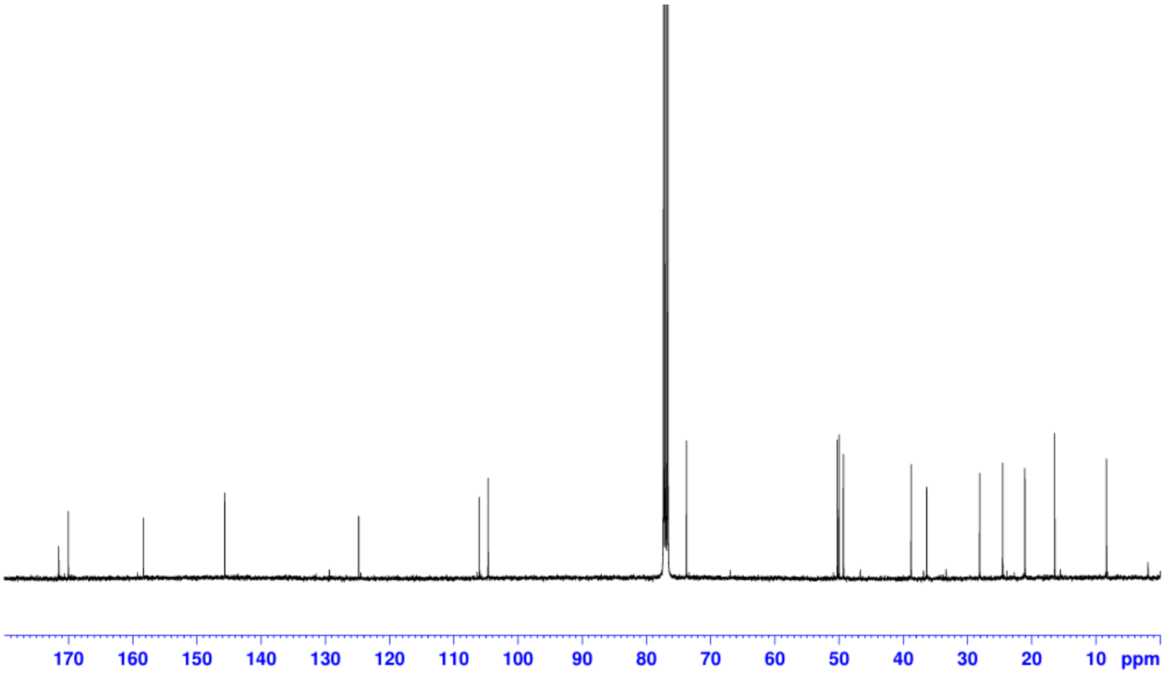

Figure S14. ^{13}C NMR spectrum of compound **7**

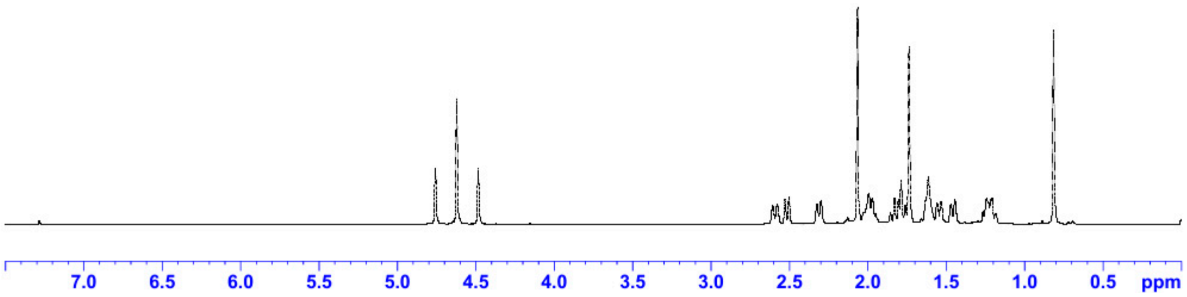

Figure S15. ^{1}H NMR spectrum of compound **8**

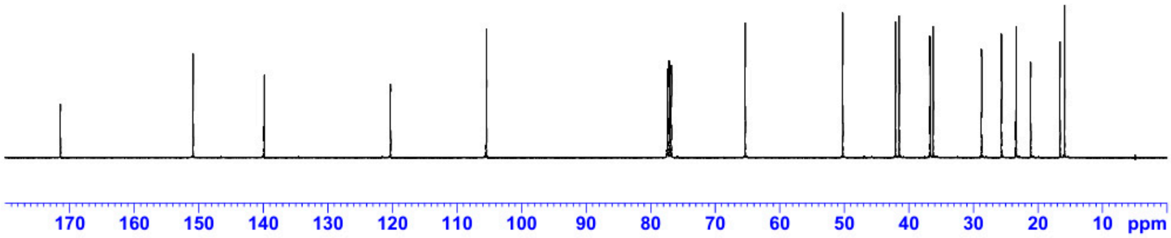

Figure S16. ^{13}C NMR spectrum of compound **8**

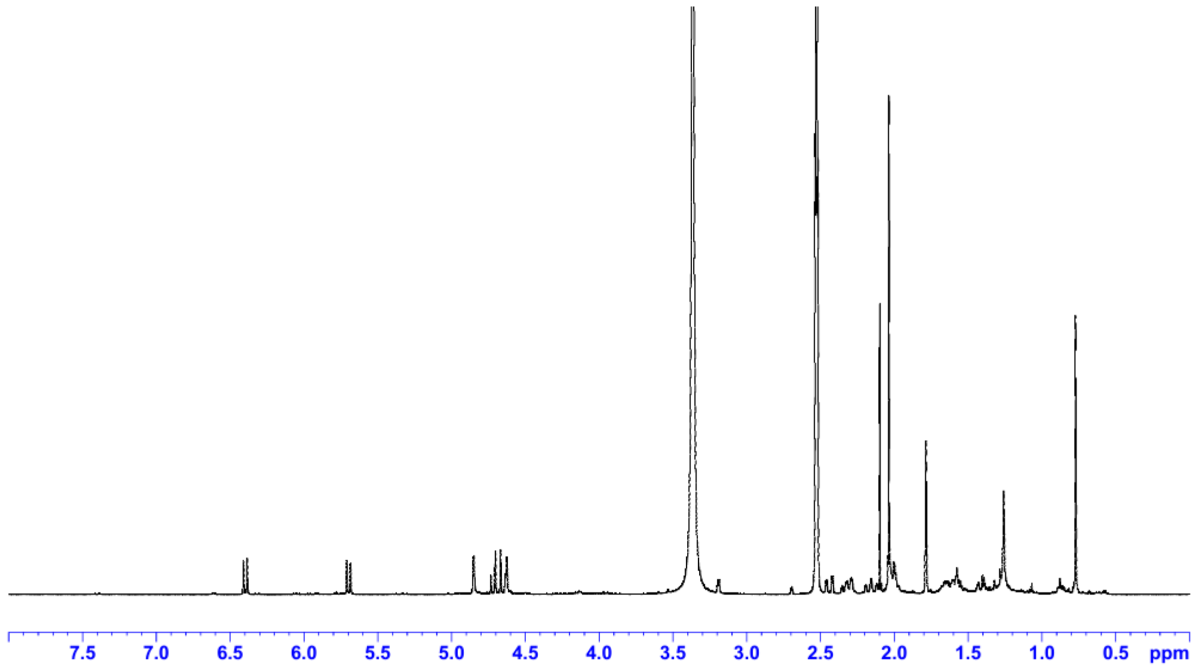

Figure S17. ^{1}H NMR spectrum of compound **9**

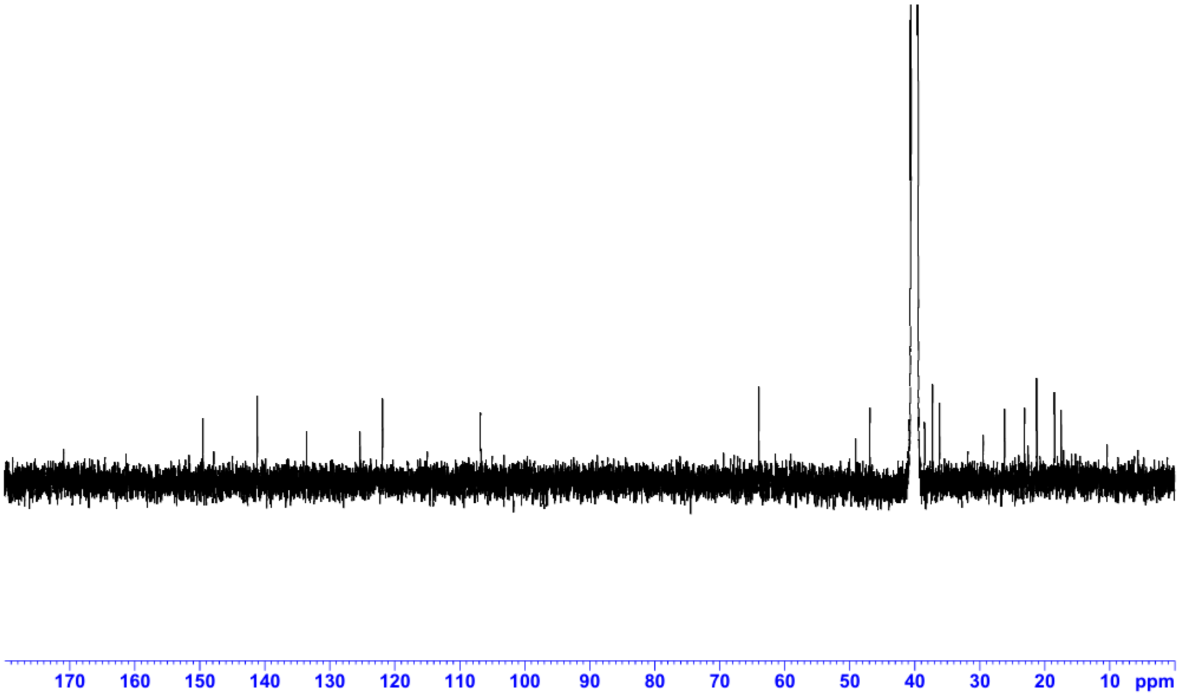

Figure S18. ^{13}C NMR spectrum of compound **9**

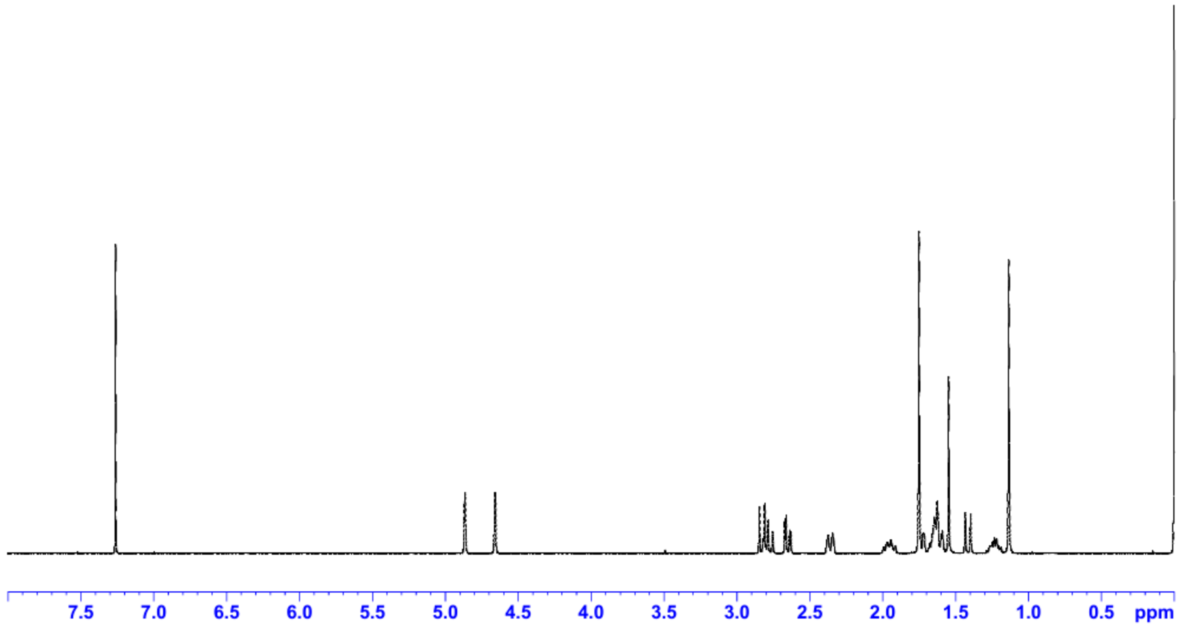

Figure S19. ¹H NMR spectrum of compound **10**

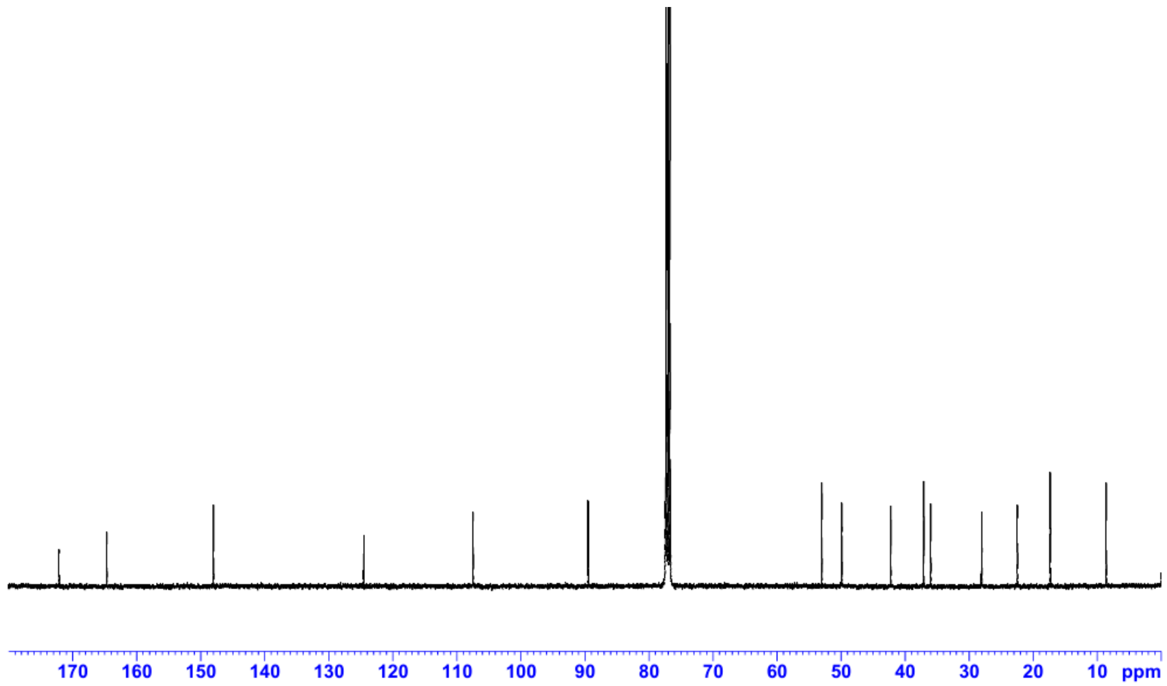

Figure S20. ¹³C NMR spectrum of compound **10**

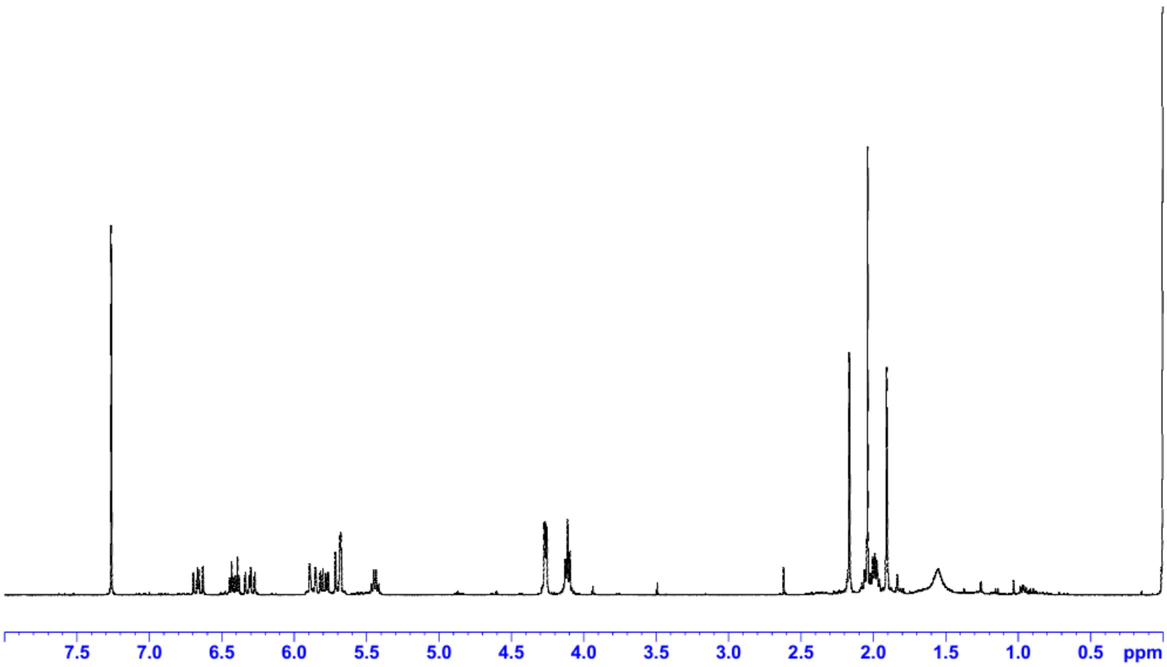

Figure S21. ^{1}H NMR spectrum of compound **11**

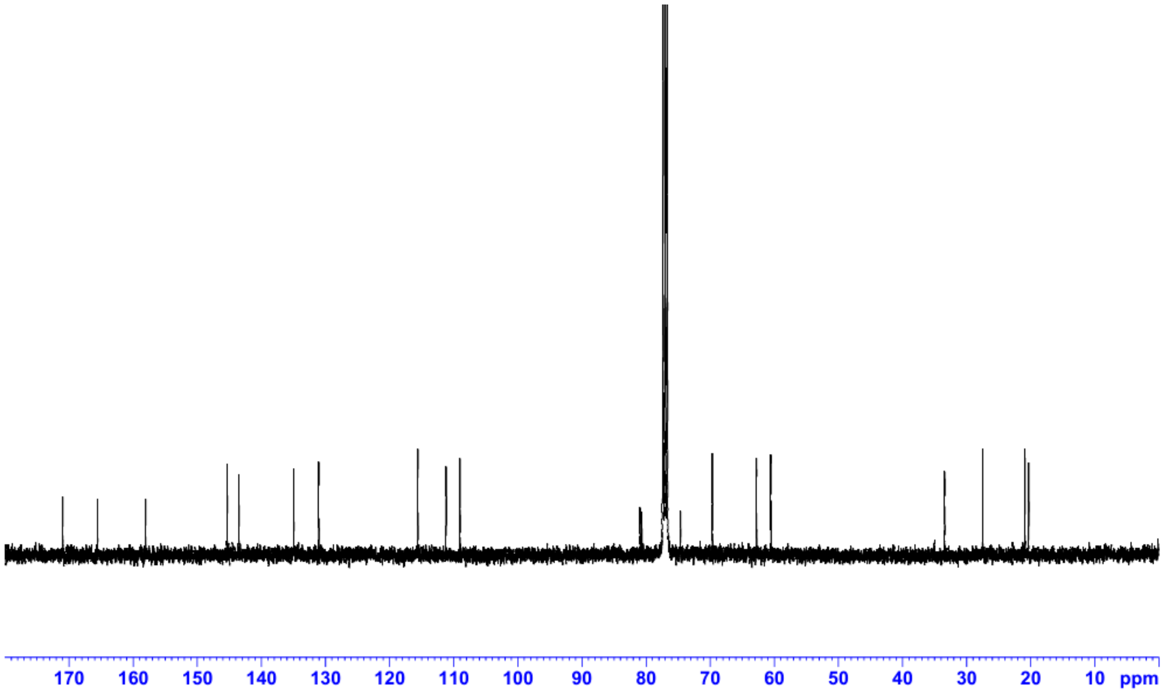

Figure S22. ^{13}C NMR spectrum of compound **11**

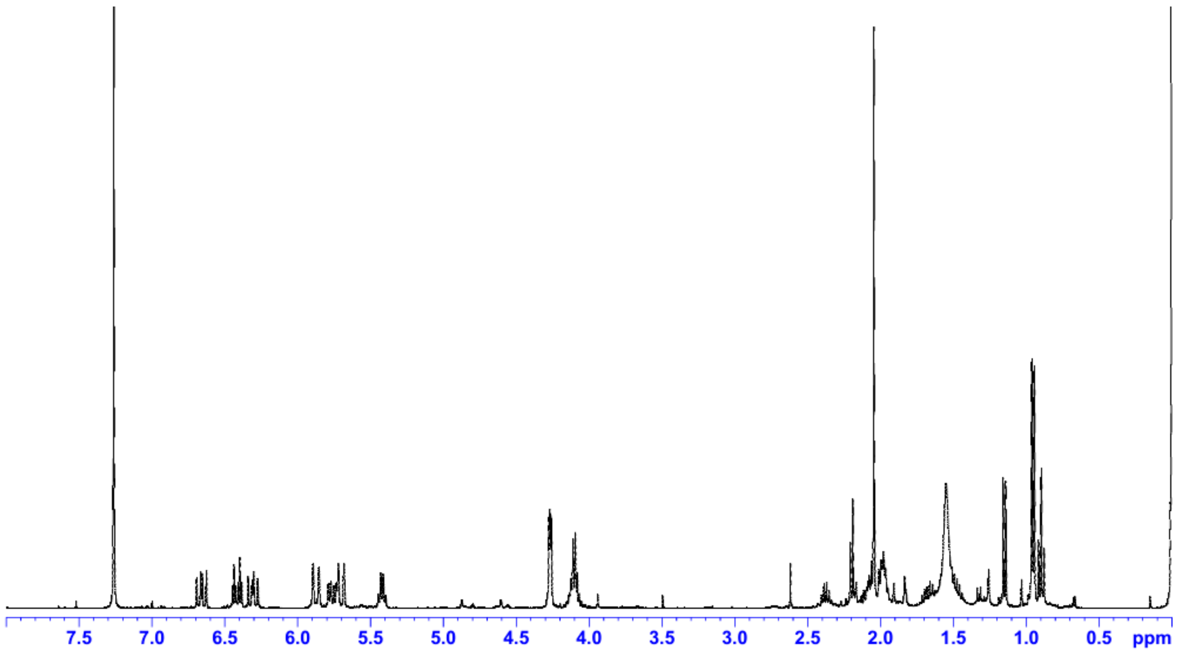

Figure S23. ^{1}H NMR spectrum of compound **12**

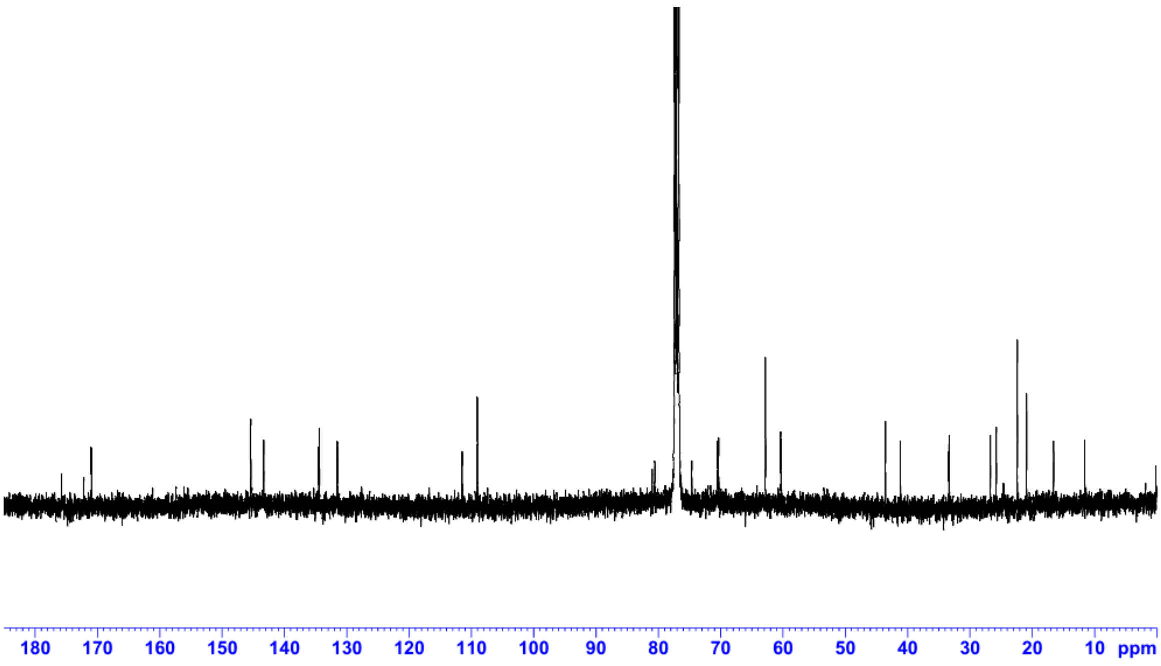

Figure S24. ^{13}C NMR spectrum of compound **12**

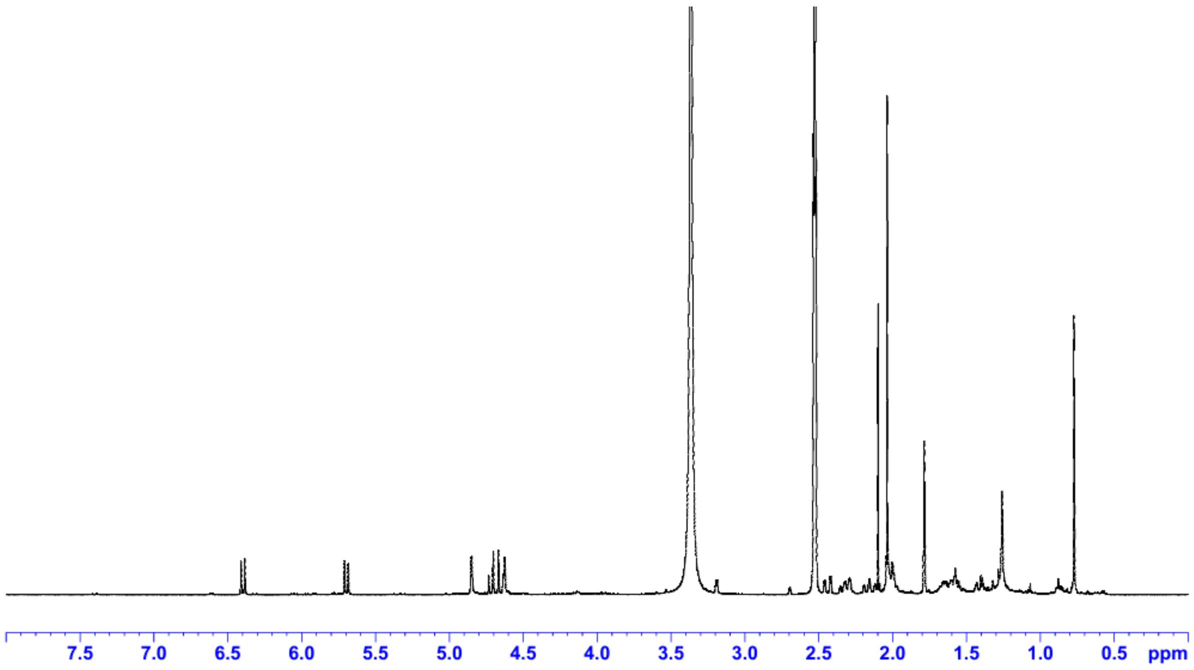

Figure S25. ^{1}H NMR spectrum of compound **13**

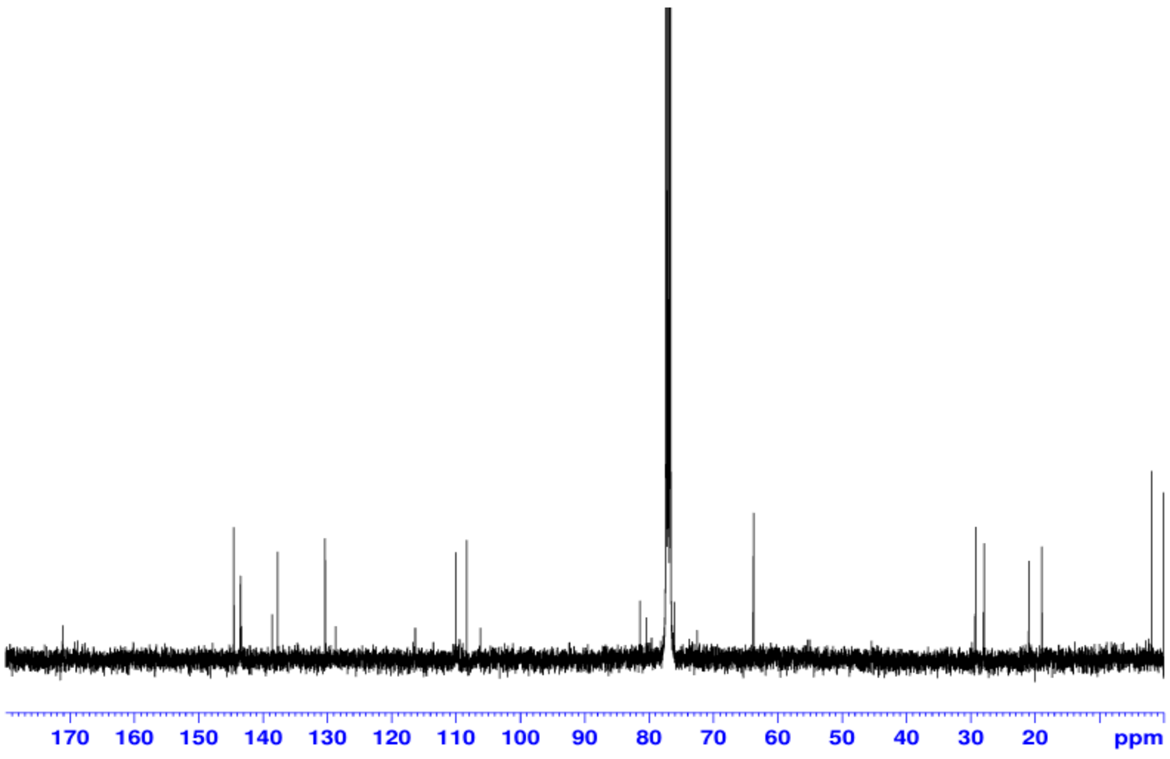

Figure S26. ^{13}C NMR spectrum of compound **13**
